# A competency framework for librarians involved in systematic reviews

**DOI:** 10.5195/jmla.2017.189

**Published:** 2017-07-01

**Authors:** Whitney A. Townsend, Patricia F. Anderson, Emily C. Ginier, Mark P. MacEachern, Kate M. Saylor, Barbara L. Shipman, Judith E. Smith

## Abstract

**Objective:**

The project identified a set of core competencies for librarians who are involved in systematic reviews.

**Methods:**

A team of seven informationists with broad systematic review experience examined existing systematic review standards, conducted a literature search, and used their own expertise to identify core competencies and skills that are necessary to undertake various roles in systematic review projects.

**Results:**

The team identified a total of six competencies for librarian involvement in systematic reviews: “Systematic review foundations,” “Process management and communication,” “Research methodology,” “Comprehensive searching,” “Data management,” and “Reporting.” Within each competency are the associated skills and knowledge pieces (indicators). Competence can be measured using an adaptation of Miller’s Pyramid for Clinical Assessment, either through self-assessment or identification of formal assessment instruments.

**Conclusions:**

The Systematic Review Competencies Framework provides a standards-based, flexible way for librarians and organizations to identify areas of competence and areas in need of development to build capacity for systematic review integration. The framework can be used to identify or develop appropriate assessment tools and to target skill development opportunities.

## BACKGROUND

Informationists and librarians have a significant role to play in the research enterprise through partnering with researchers on systematic reviews (SRs). Collaborations have become increasingly common [1], and librarian contributions extend beyond expert searching skills [2]. Expert searching, including comprehensive and replicable searches, remains a core way that librarians demonstrate their value [3]. However, through knowledge of and experience in conducting SRs, librarians can provide insight and expertise along the entire lifecycle of an SR. In many instances, researchers want to conduct their own searches but need significant assistance with resources to consult as well as search strategies to employ [4]. The consulting role can also extend into other areas, such as question refinement, processes and procedures for data extraction and management, SR methodology, and appropriate reporting for publication. In other instances, the librarian may be a full partner in the SR process by helping refine the research question, prepare and submit protocols, search the literature, manage data, and on some occasions, screen and appraise studies for inclusion before writing part of the manuscript for publication.

As informationists at the University of Michigan’s Taubman Health Sciences Library, the authors have collaborated with researchers on SRs for several years. These collaborations tend to develop with researchers who proactively invite us to participate in their projects. The number of SR-related requests continues to increase, along with demands on our time. In this environment of increasing demand, we sought to develop a set of competencies to guide our involvement in SR searching and teaching. Once created, these competencies would help build our library’s capacity by providing a framework for professional development for less experienced informationists, including new hires. Furthermore, the competencies could become a minimum set of standards to be achieved by informationists, thereby bringing more consistency to the information typically shared during consultations with SR project leads.

The Medical Library Association (MLA), other organizations, and individuals have long provided competencies and standards for health sciences librarians [5–10]. While these competencies provide excellent frameworks for skills and knowledge required in the profession as a whole, they do not explicitly address the specific structural and methodologic characteristics of SR involvement beyond broad expectations of competence in expert searching. Through a search of the literature, we found one conference poster outlining a proposed set of SR competencies for librarians [11] but did not identify additional publications. The explosion of interest in SRs among health sciences librarians—as evidenced by increases in SR-related MLA programming, the convening of the MLA Systematic Reviews Special Interest Group (SIG), and the proliferation of resources and outlets devoted to SRs—makes this the ideal time for a flexible set of guiding competencies focused on librarian involvement in SRs.

## DEVELOPMENT OF CORE COMPETENCIES

We convened in November 2015 with the express purpose of identifying and defining core competencies for informationists and librarians involved in SRs. While our primary charge was to develop competencies to build capacity and skills for local SR work, the resulting document was designed to be used by other individuals and organizations to assess readiness and skills in each competency area.

Our team purposefully included informationists at varying levels of professional experience, all of whom were integrated into SR work through instruction, consultation, and inclusion on SR teams. Five of the team members were instructors or conducted assessment for the “Systematic Reviews: Opportunities for Librarians” flipped-classroom workshop; one led the Michigan Medicine Clinical Care Guideline Development Search team; and three provided SR training in coursework for doctoral nursing candidates.

**Figure 1:**
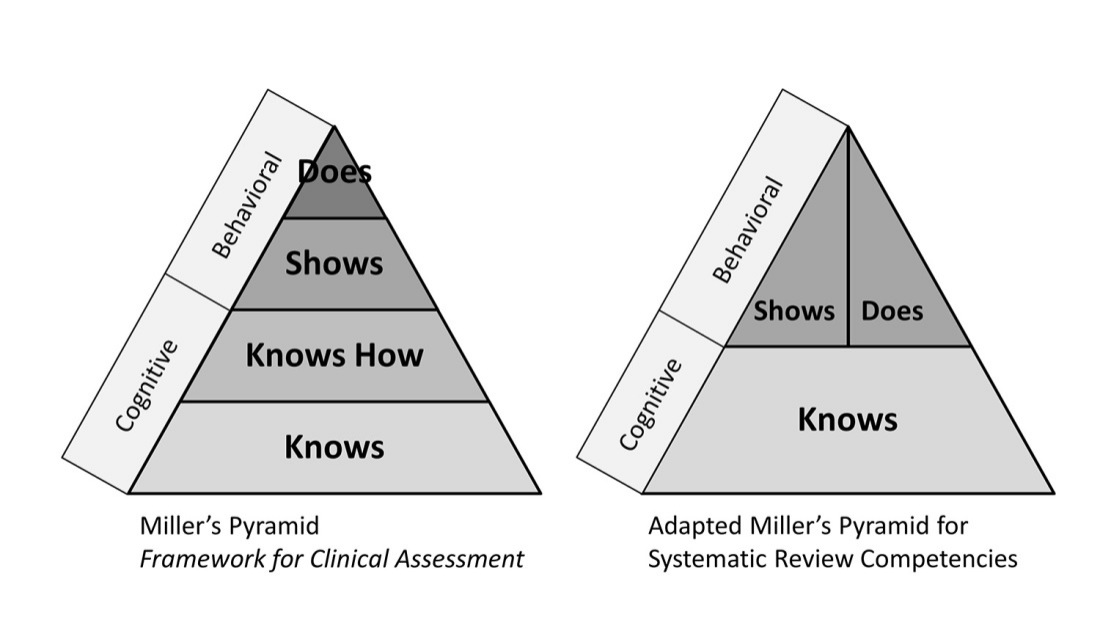
Original and adapted Miller’s Pyramid for Clinical Assessment

Team members independently generated a list of skills and knowledge items that they believed to be core components of successful integration into SR projects and teams. Next, individual team members thoroughly reviewed relevant methodology and reporting standards (Cochrane Handbook [12], Joanna Briggs Reviewer’s Manual [13], Preferred Reporting Items for Systematic Reviews and Meta-Analyses (PRISMA) [14], National Academies Standards for Systematic Reviews [15], Campbell Collaboration [16], and Centre for Reviews and Dissemination [17]) to identify additional skills and ensure consistency with established SR guidelines. We collated the results and grouped the skills and knowledge items (i.e., indicators) by theme to identify overarching competency areas, combining skills where appropriate. Finally, team subgroups defined the scope of each competency and refined the associated indicators that each competency contained. Initially, we attempted to stratify indicators by level of expertise, but it quickly became clear that the relationship of a librarian to any given SR project is not easily defined by terms like “novice,” “intermediate,” and “expert.” Instead, we adapted a version of Miller’s Pyramid for Clinical Assessment (Figure 1) to better reflect the diversity of SR-related roles that librarians take on.

## MILLER’S PYRAMID

In 1990, psychologist George Miller proposed a pyramid as a framework for assessing clinical skills [18]. The original pyramid is divided into four levels in two distinct zones: the cognitive zone (Knows, Knows How) and the behavioral zone (Shows, Does) (Figure 1). By stratifying competence in this way, educators and clinicians can identify appropriate assessment tools depending on the level of competence being measured. Mehay and Burns describe the original levels of Miller’s Pyramid as follows:

In the pyramid, the lower two levels only test cognition (or knowledge) and this is the area where inexperienced trainees (or novices) usually sit: for example, they either “know” something about a mental state examination or they “know how” to do a mental state examination. The upper two levels test behaviour: can they apply what they know into practise? Going back to the previous example: can they “show” how to do a mental state examination or do they actually “do” a mental state examination in practise? [19]

Of course, the parallels that can be drawn between Miller’s levels of competence for clinical skills and the competencies required for librarian involvement in SRs can only go so far. For our purposes, we adapted these levels to serve as modifiers for the individual indicators in each competency, allowing librarians or organizations to determine their current levels of competence and to identify skill gaps in priority competency areas. The adapted pyramid recognizes the delineation between cognitive and behavioral competence, reflecting the fact that the instructional and consultative roles that librarians often fill require a level of skill equal to, although different from, the performance of those skills as part of an SR team (Figure 1).

## SYSTEMATIC REVIEW COMPETENCY FRAMEWORK

Individual librarians’ involvement in and integration with SRs can vary widely depending on their environments, their personal goals, and their institutions’ commitment to and capacity for integration into different facets of the SR process. The resulting competencies represent a broad set of areas in which librarians can develop skills and expertise to support their integration with SRs in their institutions and as part of their professional practice. Each competency has specific knowledge items and skills that serve as indicators of competence. Individual librarians may have differing levels or types of competence depending on their roles on a given SR team and their organizations’ priorities and culture. Whereas one librarian may regularly serve as a team methodologist, another may serve as a consultant for search strategy development. By incorporating the levels of the adapted Miller’s pyramid into the framework, the indicator “Protocol development, including question refinement, inclusion/exclusion criteria, data management plan, and protocol registration” in the “Research methodology” competency can be modified to identify three distinct levels of competence:

- **Knows:** Knows about and understands [protocol development], including question refinement, inclusion/exclusion criteria, data management plan, and protocol registration
- **Shows:** Applies knowledge [of protocol development] through instruction, consultation, or referral to resources
- **Does:** Applies knowledge [of protocol development] consistently as a part of SR teams

The Systematic Review Competency Framework (Table 1) does not propose required levels for each indicator, as individual librarians (even within the same organization) may have differing levels of involvement in or prioritization of SRs as part of their positions. Rather, the framework is designed to help librarians and their organizations to identify their current cognitive or behavioral levels in each competency area and to identify development opportunities to build personal or institutional skills and capacity for SR work that is consistent with recognized SR standards and practices. Similarly, the framework does not provide specific assessment tools to measure level of competence but allows personal and institutional interpretation and identification of appropriate assessments, if desired.

**Table 1:** Systematic Review Competencies Framework

Competency	Scope note	Indicator	Cognitive	Behavioral	
			Knows (knows about and understands)	Shows (applies knowledge through instruction, consultation, or referral to resources)	Does (applies knowledge consistently as a part of SR teams)
Systematic review (SR) foundations	How and why SRs are used in the health sciences and how to effectively find SRs	Application of SRs in the health sciences (e.g., clinical care, policy making)			
		Resources and strategies to limit searches to find SRs			
		Methodological distinctions between SRs and other types of literature reviews and primary studies			
Process management and communication	Resources and skills involved in SR team communication and effective project management	Negotiation of librarian role in SR process (e.g., conducting the searches versus consultative role)			
		Authorship and acknowledgment criteria and responsibilities			
		Typical SR timeline, team composition, and associated tasks			
		Available technologies and avenues for team communication, document sharing, and document archiving (e.g., email, cloud services, conference calls, video conferencing, in-person)			
		Strategies to keep collaborators apprised about search progress and timeline			
		Communication of nuances of exhaustive literature searches and SR searches			
Research methodology	Ability to comply with and advise teams on SR standards and best practices	Matching of research question to an appropriate review type			
		Core SR methodology and reporting standards, guides, and handbooks			
		Protocol development, including question refinement, inclusion/exclusion criteria, data management plan, and protocol registration			
		Searching (see “Comprehensive searching” competency)			
		Data management (see “Data management” competency)			
		Critical appraisal of SR quality			
		Study selection processes, including inter-rater reliability, risk of bias, and critical appraisal			
Comprehensive searching	Ability to construct and document replicable search strategies in appropriate literature databases and other information resources	Conduct of preliminary searches to confirm the need for an SR			
		Database selection appropriate to the research question, including grey literature resources			
		Techniques and strategies for keyword and controlled vocabulary search term generation			
		Search filters, including validation and sources			
		Creation of comprehensive search strategies in multiple databases using advanced search techniques			
		Search strategy validation techniques, including use of sentinel articles			
		Techniques and tools to save searches, set auto-alerts, and update searches			
		Transparent and complete documentation procedures for replicable searches			
Data management	Processes, tools, and skills involved in using data and ensuring data integrity, archiving, and tracking for the SR process	Citation management software (e.g., EndNote, RefWorks, Mendeley)			
		SR software, including strengths, limits, and uses (e.g., Distiller, Covidence, Rayyan)			
		Data extraction tools and forms			
		Appropriate data and process archiving, including version tracking and PRISMA data collection			
Reporting	Ability to communicate literature search methods and results according to established standards so that they are suitable for publication and are replicable	Reporting standards associated with the literature search			
		Transformation of final search strategies into replicable format for publication (e.g., online appendix or supplement)			
		Communicating the precise search process for publication, including the essential search-related information listed in PRISMA			

## ASSESSMENT OF COMPETENCIES

Although we do not recommend specific tools to assess SR competencies in this article, the adapted Miller’s pyramid can assist individuals and organizations in developing formal or informal measures of competence. In Miller’s original pyramid, competence at the cognitive levels (Knows, Knows How) is best assessed using traditional true/false, multiple choice, and essay-type questions that demonstrate acquisition of knowledge but do not demonstrate application of that knowledge in practice. By contrast, competence at the behavioral levels (Shows, Does) is assessed through simulation activities and direct observation [19].

In the context of the adapted pyramid, some suggested measures could include:

- **Knows**
  – Goal: knowledge gathering and interpretation
  – Assessment methods: Completion of formal SR training program; multiple-choice or essay questions for competency indicators
- **Shows**
  – Goal: Demonstration of knowledge
  – Assessment methods: Observed instruction and consultations
- **Does**
  – Goal: Knowledge integrated into practice
  – Assessment methods: Published or working documentation for specific SR projects

As examples, the Preferred Reporting for Electronic Search Strategies (PRESS) form [20] for search strategies could be used to assess the Does level for many of the indicators for the “Comprehensive searching” competency, and comparing published or drafted search methods to PRISMA standards [14] could do the same for indicators for the “Reporting” competency. When framed this way, expectations of librarians learning about or being involved in SRs are clear and measurable with the development of appropriate assessment tools.

## FUTURE DIRECTIONS

The Systematic Review Competencies Framework highlights areas of expertise in which librarians should achieve cognitive and behavioral competence, depending on their environment and professional goals. The framework can also guide librarians in their professional development and training by identifying cognitive or behavioral knowledge gaps. For libraries considering formalizing or expanding an SR program or service, the framework may be used to ensure appropriate capacity among team members. Administrators can use these competencies to set continuing education and professional development priorities during strategic planning [11].

We understand that the MLA Systematic Review SIG began the planning process to develop a set of SR competencies in June 2016. We anticipate a high level of agreement, as well as potential differences, between our independently developed documents. We hope that our Systematic Review Competencies Framework and the MLA Systematic Review SIG document encourage continued discussion of the cognitive and behavioral skills that contribute to successful librarian involvement in SRs. We anticipate that these competencies and indicators will continue to evolve as librarians take on new roles with SRs and that they can be adapted to reflect the unique needs of different review types that require systematic searches and data management.
